# Effect of photobiomodulation and exercise on early remodeling of the Achilles tendon in streptozotocin-induced diabetic rats

**DOI:** 10.1371/journal.pone.0211643

**Published:** 2019-02-04

**Authors:** Anderson Rodrigues de Oliveira, Flávio Santos da Silva, Raul Hernandes Bortolin, Dáfiny Emanuele da Silva Marques, Gracielle Vieira Ramos, Rita C. Marqueti, Naisandra Bezerra da Silva, Karina Carla de Paula Medeiros, Márcio Assolin Corrêa, João Paulo Matos Santos Lima, Adriana Augusto de Rezende, Paul W. Ackermann, Bento J. Abreu, Wouber Hérickson de Brito Vieira

**Affiliations:** 1 Department of Physical Therapy, Federal University of Rio Grande do Norte, Natal, Brazil; 2 Department of Health Sciences, Federal University of the Semiarid Region, Mossoró, Brazil; 3 Department of Clinical and Toxicological Analysis, Federal University of Rio Grande do Norte, Natal, Brazil; 4 Department of Morphology, Federal University of Rio Grande do Norte, Natal, Brazil; 5 Universidade Paulista (UNIP), Brasília, Brazil; 6 University of Brasília (UnB), Brasília, Brazil; 7 Department of Physics, Federal University of Rio Grande do Norte, Natal, Brazil; 8 Department of Biochemistry, Federal University of Rio Grande do Norte, Natal, Brazil; 9 Department of Molecular Medicine and Surgery, Karolinska University Hospital, Karolinska Institutet, Stockholm, Sweden; Massachusetts General Hospital, UNITED STATES

## Abstract

The aim of this study was to compare the treatment effects of laser photobiomodulation (LPBM) therapy and aerobic exercise on the biomechanical properties, tissue morphology and the expression of tendon matrix molecules during early remodeling of Achilles tendon (AT) injury in diabetic rats. Animals were randomly assigned to five groups: injured non diabetic (I, n = 15), injured diabetic (ID, n = 15), injured diabetic plus LPBM (IDL, n = 16), injured diabetic plus aerobic exercise (IDE, n = 16) and injured diabetic plus aerobic exercise and LPBM (IDEAL, n = 17). Type 1 diabetes was induced via a single intravenous injection of Streptozotocin at a dose of 40 mg/kg. A partial tenotomy was performed in the right AT. LPBM was performed with an indium-gallium-aluminum-phosphide 660 nm 10 mW laser device (spot size 0.04 cm^2^, power density 250 mW/cm^2^, irradiation duration 16 s, energy 0.16 J, energy density 4 J/cm^2^) on alternate days for a total of 9 sessions over 3 weeks (total energy 1.44 J), using a stationary contact technique to a single point over the dorsal aspect of the AT. Moderate aerobic exercise was performed on a motorized treadmill (velocity 9 m/min for 60 minutes). At 3 weeks post-injury, biomechanical analyzes as well as assessment of fibroblast number and orientation were performed. Collagen 1 (Col1) and 3 (Col3) and matrix metalloproteinases (MMPs) -3 and 13 protein distributions were studied by immunohistochemistry; while Col1 and Col3 and MMP-2 and 9 gene expression were assessed by quantitative RT-PCR (qRT-PCR). IDEAL exhibited significant increases in several biomechanical parameters in comparison to the other groups. Moreover, IDEAL presented stronger Col1 immunoreactivity when compared to ID, and weaker Col3 immunoreactivity than IDE. Both IDL and IDEAL demonstrated weaker expression of MMP-3 in comparison to I, while IDL presented no expression of MMP-13 when compared to ID. ID, IDL and IDE showed an increased number of fibroblasts in comparison to I, while IDEAL decreased the number of these cells in comparison to ID and IDE. IDL and IDEAL groups exhibited decreased angular dispersion among the fibroblasts when compared to I. The gene expression results showed that IDE demonstrated a downregulation in *Col1* mRNA expression in comparison to I and ID. IDEAL demonstrated upregulation of *Col1* mRNA expression when compared to IDL or IDE alone and increased *MMP-2* expression when compared to IDL and IDE. *MMP-9* expression was upregulated in IDEAL when compared to I, IDL and IDE. Our results suggest a beneficial interaction of combining both treatment strategies i.e., aerobic exercise and LPBM, on the biomechanical properties, tissue morphology and the expression of matrix molecules in diabetic tendons.

## Introduction

Diabetes Mellitus (DM) is a complex metabolic disease characterized by chronic hyperglycemia which is responsible for several long term systemic complications [1]. In the last years, a growing body of evidence has demonstrated the association between DM and tendinopathy [2,3]. Indeed, this painful connective tissue disorder can affect up to 60% of diabetic patients [4] and cause considerable disability, possibly due to compromised regenerative and healing capability [5].

While the pathogenesis of diabetic tendinopathy remains to be fully detailed [6], it is well known that the diabetic tendon exhibits a wide range of cellular, morphological, biomechanical and expressional alterations [1,7]. Among these alterations, collagen fiber disorganization and altered expression of key extracellular matrix (ECM) proteins, such as matrix metalloproteinases (MMPs) -2, -3, -9 and -13 [8] were previously reported as possible mechanisms for the impaired tendon healing in DM as they can lead to decreased degradation of matrix proteins and impaired tissue remodeling [9].

Different modalities of laser photobiomulation (LPBM) therapy [10–12] and physical activity [13–15] have been used to improve normal and diabetic tendon healing in animals and humans. LPBM, for instance, has been demonstrated to modulate inflammation and reduce edema in tendon healing [16,17] in addition to alter gene expression of several MMPs in diabetic wounds and to enhance collagen production [18]. On the other hand, moderate aerobic exercise has been shown to attenuate tendinous complications in diabetes [15], restore tendon biomechanical properties [14], enhance collagen synthesis and increase the organization of the tendon matrix [19]. However, the underlying mechanisms by which LPBM and exercise enhance tendon healing remain to be determined, especially in DM.

Thus, the aim of the present study was to compare the treatment effects of LPBM and aerobic exercise on the morphology, mechanical properties and on gene and / or immunoexpression of type 1 collagen (Col1) and type 3 collagen (Col3) and MMPs -2, -3, -9 and -13 after 3 weeks of Achilles tendon (AT) injury in chemically-induced type 1 diabetes in rats. To the best of our knowledge, this is the first work that investigated the association of both types of treatment on the proliferative / early remodeling of the injured diabetic AT.

## Materials and methods

### Animals and study design

Seventy-nine 60-days-old male Wistar rats weighing 220 ± 20 g were obtained from the animal care facility of the Federal University of Rio Grande do Norte, Brazil. The animals were housed in climate-controlled conditions (12 h light/dark cycle, 22–24°C and 50–60% relative humidity), with food and water *ad libitum* provided during the entire experimental period. All animal experiments were approved by the Ethics Committee at the Federal University of Rio Grande do Norte (protocol number 038/2014). All procedures were carried out in strict accordance with the recommendations from the Guide for the Care and Use of Laboratory Animals of the National Institutes of Health. All efforts were made to minimize animal suffering, including gentle handling, daily cage cleaning and monitoring. Animals were randomly assigned to five groups: injured non diabetic (I, n = 15), injured diabetic (ID, n = 15), injured diabetic plus LPBM (IDL, n = 16), injured diabetic plus aerobic exercise (IDE, n = 16) and injured diabetic plus aerobic exercise and LPBM (IDEAL, n = 17).

Experimental DM was induced via a single intravenous injection of streptozotocin (STZ, Sigma-Aldrich, St. Louis, MO, USA) dissolved in freshly prepared sodium citrate buffer (0.1 M, pH 4.5) at a dose of 40 mg/kg [20]. Equal volumes of vehicle were injected into the non-diabetic rats. On the fifth day after the induction, the blood samples were collected from tail vein and glycaemia was assayed using an Accu-Chek Advantage glucometer (Roche Diagnostics, Indianapolis, IN, USA). Animals with blood glucose concentrations > 250 mg/dL were considered diabetic. Moreover, polydipsia, polyphagia and polyuria could be observed in all diabetic rats. Blood glucose was also observed at the end of the experimental period.

After one week of STZ-induced diabetes, rats of all groups were anesthetized with isoflurane (2–3%). The AT injury was performed following the protocol described elsewhere [21–23]. Briefly, after surgical exposure of the tendon, a partial tenotomy was performed in the middle third of the right AT using an 18-gauge needle (S1 Fig). The skin was then sutured with nylon 4.0 wire and the animals were returned to their cages for resting. Animals were unrestricted inside their cages. After 21 days of AT injury, animals were euthanized with a solution of xylazine (12 mg/Kg) and ketamine hydrochloride (90 mg/kg), 0.10 ml for each 100 g of weight, and ATs were harvested and stored for mechanical testing, immunohistochemistry and gene expression procedures.

### LPBM protocol

An indium-gallium-aluminum-phosphide laser (MMoptics, São Carlos, SP, Brasil) was used in this work with a power output of 10 mW (power density of 250 mW/cm^2^), beam area of 0.04 cm^2^ (according to the manufacturer specification) and wavelength (λ) of 660 nm. The laser parameters and application protocol were chosen according to previous studies that showed their effectiveness for tendon healing [24,25]. Energy density was 4 J/cm^2^, with a total energy of 0.16 J and 16 s of exposure time. The application was performed by a trained researcher, with the laser probe in direct contact to a single point over the dorsal aspect of the AT of IDL and IDEAL rats. The LPBM therapy was started on the following day after AT injury induction and consisted of 9 sessions (3 times per week) performed on alternate days, during 3 weeks. For the IDEAL group, the alternate-day irradiations were performed immediately after the exercise session, given that mechanical and metabolic stress have been suggested to enhance LPBM effects [26].

### Exercise protocol

A protocol of moderate aerobic exercise was performed by using a motorized treadmill (Insight, São Paulo, SP, Brazil), according to a previous work [14]. Briefly, the animals of the groups that performed physical activity (IDE and IDEAL) underwent a period of familiarization to the treadmill for two weeks before the beginning of the experiments. On the first two days, animals were kept on the turned off treadmill for 60 minutes. From third to last day of the adaptation period, running speed and duration progressed from 4 m/min for 12 minutes to 9 m/min for 60 minutes. Then, exercise protocol was initiated one day after the AT injury. Each exercise session was set to 9 m/min, for 60 min, and occurred 5 days a week during 3 weeks, totaling 15 sessions (S2 Fig). Each daily session was preceded by warm-up period and was followed by cool-down period. During the experiments, the animals were monitored for problems of weight loss, food intake or any sign of discomfort. After the exercise session, the animals of the IDE and IDEAL groups were allocated in their respective cages for resting.

### Mechanical testing

For mechanical testing, specimens of each group (I, n = 6; ID, n = 6; IDL, n = 6; IDE, n = 7; IDEAL, n = 8) were retrieved from the freezer and allowed to thaw at room temperature 6 h before testing, as described elsewhere [27]. Then, soft tissue was removed and ATs were attached to a metal connector (2.5 x 3.5 cm) at each extremity and pinned to a conventional mechanical testing machine equipped with a load cell of 500 N. Tendon thickness and width were measured with a digital caliper and then specimens were pulled to failure at a speed of 0.1 mm/s. The evaluated parameters were: ultimate load (N), ultimate elongation (mm), stiffness (N/mm), absorbed energy (J), cross-sectional area (mm^2^), ultimate strength (MPa), ultimate strain (%), elastic modulus (MPa) and absorbed energy/CSA (J/mm^2^).

### Immunohistochemistry

For assessing the expression of key ECM proteins, 4 tendons of each group were harvested and fixed in Zamboni’s fixative consisting of paraformaldehyde (4%) in 0.2 mol/l Sörensen phosphate buffer, pH 7.3, containing picric acid (0.2%) at 4°C for 48 h. After this period, tissues were soaked in sucrose (20%) in 0.1 mol/l Sörensen phosphate buffer, pH 7.2, containing sodium azide and bacitracin (Sigma Chemicals, St. Louis, Mo., USA). ATs were longitudinally sectioned by using a Leitz 1720 cryostat (Ernst Leitz,Wetzlar, Germany) and 12 μm thickness sections were mounted on SuperFrost/Plus slides. Three sections from the injured right ATs from each group were immunostained with antiserum against Col1, Col3, MMP-3 and MMP-13. Possible non-specific binding was minimized by pre-incubating the sections in 5% normal goat serum for 30 min. After, the sections were incubated overnight with specific antisera to Col1 (1:200), Col3 (1:200), MMP-3 (1:200) and MMP-13 (1:100, Santa Cruz Biotechnology, Santa Cruz, Calif., USA). Sections were rinsed in PBS (3 × 5 min) and incubated for 30 min at room temperature with secondary antibodies; goat anti-rabbit or donkey anti-goat (1:250, Vector Laboratories, Burlingame, Calif., USA). Sections were then washed with PBS (3×5 min) and incubated with ABC reagent for 30 min at room temperature. Diaminobenzidine (DAB) chromogen (Vector Laboratories) and counterstaining with Hematoxylin QS (Vector Laboratories) were then applicated. Next step consisted of section’s dehydration (70%, 95%, and then 99%) with ethanol. Control sections were then stained with the primary antisera being either omitted or pre-adsorbed with the corresponding ligand peptides in order to confirm staining’s specificity. Images from every injury site were captured at 200-fold magnification by a video camera attached to a microscope (Nikon Eclipse 80i, International Institute of Neurosciences Edmond and Lily Safra, Macaíba, RN, Brazil) and then stored for further analysis. Quantification of protein expression was performed on ImageJ software (NIH, Bethesda, MD, USA; http://rsb.info.nih.gov/nih-image/) using the IHC Profiler plugin, according to the protocol of Varghese and colleagues [28].

### Automated morphometry

Fibroblast number and orientation were analyzed in the same fields of view as those assessed for immunoexpression using digital image processing techniques on ImageJ, adapting the method described by Erisken et al [29]. Briefly, a custom ImageJ macro was written to retain only the spindle-shaped basophilic profiles (i.e., fibroblast nuclei). The ImageJ built-in function ‘Analyze Particles’ was then used to count fibroblast nuclei and measure their individual orientations in each histological field. Tissue organization was appraised by calculating the circular standard deviation (the circular statistical equivalent of the standard deviation for linear data) of the angular distribution of the fibroblast nuclei in the images. The smaller the circular standard deviation, the more aligned the nuclei [29].

### RNA extraction and reverse transcriptase quantitative polymerase chain reaction (RT-qPCR)

Five tendons of each group were homogenized in a tube containing five stainless steel balls of 2.3 mm in diameter (BioSpec Products, Bartlesville, OK), and one silicon-carbide sharp particle of 1 mm (BioSpec Products), by which they were shaken in a FastPrep-24 instrument (MP Biomedicals). In order to obtain complete tissue homogenization, the shaking process was repeated seven times with ice cooling between each shaking step. Total RNAs were extracted using the RNeasy Fibrous Tissue Mini Kit (Qiagen, Valencia, CA), according to the manufacturer’s protocol and RNase-Free DNase Set enzyme (Qiagen, Valencia, CA) was used for DNA digestion. RNA integrity was assessed by electrophoresis in 1.0% agarose gels with MOPS [3-(N-morpholino) propanesulfonic acid] buffer, and RNA was quantitated using a NanoDrop ND-1000 spectrophotometer (Thermo Scientific, Wilmington, DE, USA). cDNA was synthesized using 500 ng of total RNA and Sensiscript Kit reverse transcriptase (Qiagen, Valencia,CA) and 1 μM poly-dT (Invitrogen), according to the manufacturer’s protocol (Qiagen, Valencia, CA). RT-qPCR was performed using the TaqMan assay with the genes Col1 (Rn01463848_m1), Col3 (Rn01437681_m1), MMP2 (Rn01538170_m1), MMP9 (Rn01775763_g1) and glyceraldehyde-3-phosphate dehydrogenase (GAPDH, Rn00579162_m1) (Applied Biosystems). GAPDH was used as the reference gene due to greater stability if compared to RPLP0 and β-actin, as observed previously [12]. PCR assays were carried out in 96-well plates using a 7500 Fast Real-Time PCR System (Applied Biosystems). Transcript abundances were normalized to the expression of GAPDH and expressed as delta Ct values (ΔCt = Ct^GAPDH^–Ct^target^). Higher ΔCt values indicate a higher relative expression of the target genes [30].

### Statistical analysis

Statistical calculations were performed with GraphPad Prism 5.0 (GraphPad Software, CA, USA). Differences between groups were tested using one-way analysis of variance with Tukey's post hoc test. Data showing non-Gaussian distribution were compared using Kruskal–Wallis test followed by Dunn's multiple-comparison test. Paired t-test was used to check within-group differences over the experimental period regarding body weight and blood glucose levels. The p-value < 0.05 was considered as statistically significant.

## Results

### Blood glucose, body weight and tendon thickness

Table 1 presents the data obtained for blood glucose, body weight and AT thickness. As expected, blood glucose concentrations in diabetic groups were higher than those in control groups (p < 0.05). Final blood glucose concentrations were not statistically different to initial concentrations with the exception of IDEAL, which exhibited decreased final blood glucose levels. Hyperglycemia was also associated with polyphagia, polydipsia, and polyuria (data not shown) in the diabetic rats, indicating that experimental diabetes was successfully induced. The baseline body weight at the beginning of the study was not statistically different between non-diabetic and diabetic groups (average 253 ± 15 g). After the 4-week experimental period, however, all diabetic groups demonstrated decreased body weight (p < 0.05).

**Table 1:** Effects of LPBM and exercise on blood glucose (BG), body weight (BW) and AT thickness.

	I	ID	IDL	IDE	IDEAL
**Initial BG (mg/dL)**	144.00 ± 8.65	529.50 ± 23.47^a^	584.92 ± 10.04^a^	547.50 ± 20.51^a^	551.25 ± 12.13^a^
**Final BG (mg/dL)**	155.17 ± 11.24	550.25 ± 18.54^a^	541.77 ± 29.02^a^	518.61 ± 15.44^a^	478.40 ± 26.81^a^^#^
**Initial BW (g)**	247.58 ± 6.79	258.31 ± 3.41	257.15 ± 2.56	252.56 ± 4.89	245.55 ± 2.49
**Final BW (g)**	284.33 ± 6.76^#^	213.00 ± 8.91^a^^#^	232.00 ± 6.60^a^^#^	229.94 ± 7.65^a^^#^	216.15 ± 4.57^a^^#^
**AT thickness (mm)**	1.73 ± 0.23	1.24 ± 0.07	1.28 ± 0.11	1.57 ± 0.09	1.07 ± 0.02^a^
**AT thickness/BW (mm/g)**	6.08 ± 0.81	5.82 ± 0.35	5.53 ± 0.49	6.84 ± 0.39	4.97 ± 0.09^d^

I, injured (n = 15); ID, injured diabetic (n = 15); IDL, injured diabetic plus LPBM (n = 16); IDE, injured diabetic plus aerobic

exercise (n = 16); IDEAL, injured diabetic plus aerobic exercise and LPBM (n = 17).

^**#**^*P*<0.05 vs initial values.

^a^*P* < 0.05 vs I group

^d^*P* < 0.05 vs IDE group.

Tendons of IDEAL demonstrated a decrease in thickness in comparison with I (P < 0.05). When AT thickness was normalized by body weight, IDEAL demonstrated decreased values only when compared to IDE (P < 0.05).

### Mechanical testing data

**Fig 1:**
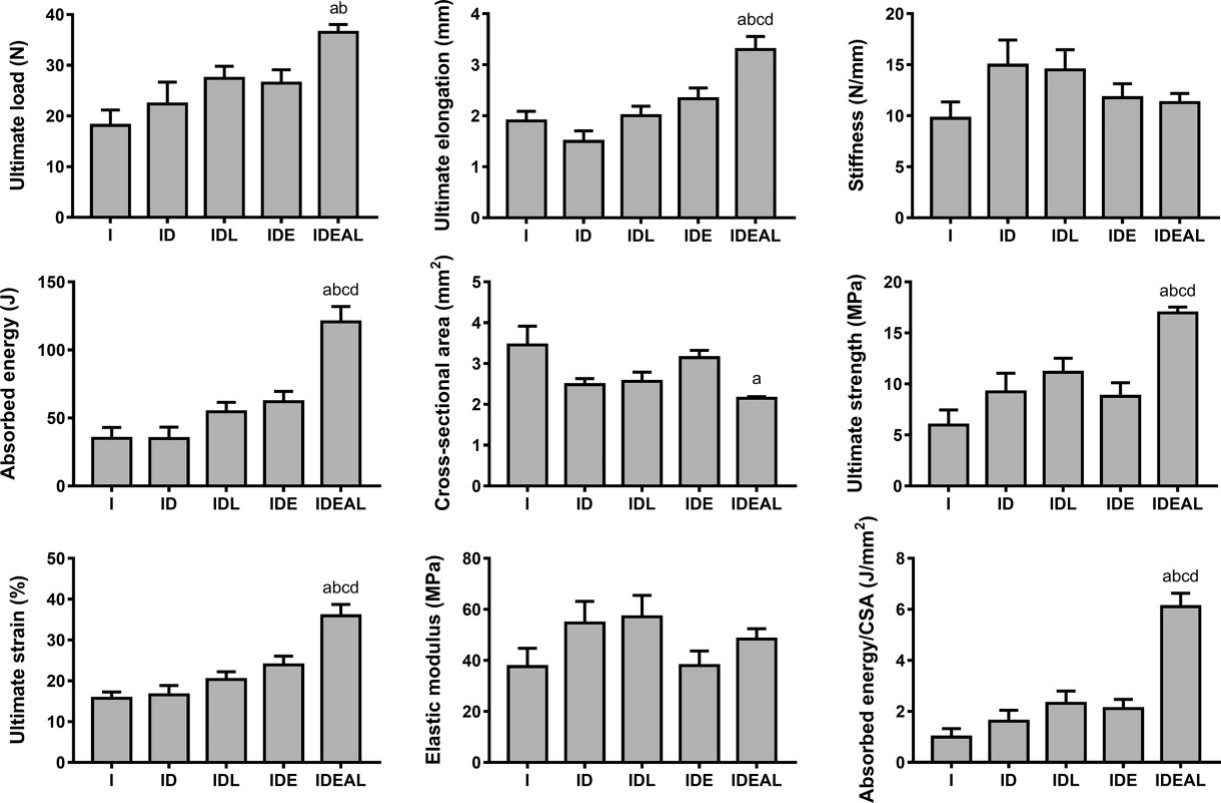
Effects of LPBM and exercise on mechanical properties and cross sectional area (CSA) of the AT. I, injured (n = 6); ID, injured diabetic (n = 6); IDL, injured diabetic plus LPBM (n = 6); IDE, injured diabetic plus aerobic exercise (n = 7); IDEAL, injured diabetic plus aerobic exercise and LPBM (n = 8). ^a^P<0.05 vs I group; ^b^P<0.05 vs ID group; ^c^P<0.05 vs IDL group; ^d^P<0.05 vs IDE group.

Fig 1 summarizes the structural and biomechanical behavior of all groups tested. Overall, there were no significant differences between I and ID (P > 0.05) for all parameters analyzed. LPBM or exercise as single interventions were not able to promote significant biomechanical alterations in comparison to I and ID (P > 0.05). When LPBM was combined with exercise, however, significant increases of ultimate elongation, absorbed energy, ultimate strength, ultimate strain and absorbed energy/CSA in comparison to the other groups was observed (P < 0.05). Moreover, IDEAL demonstrated increased ultimate load when compared to I and ID (P < 0.05); and showed decreased cross sectional area in comparison to I (P < 0.05). The different groups did not exhibit statistically significant differences in elastic modulus and stiffness (P > 0.05).

### Extracellular matrix protein expression

**Fig 2:**
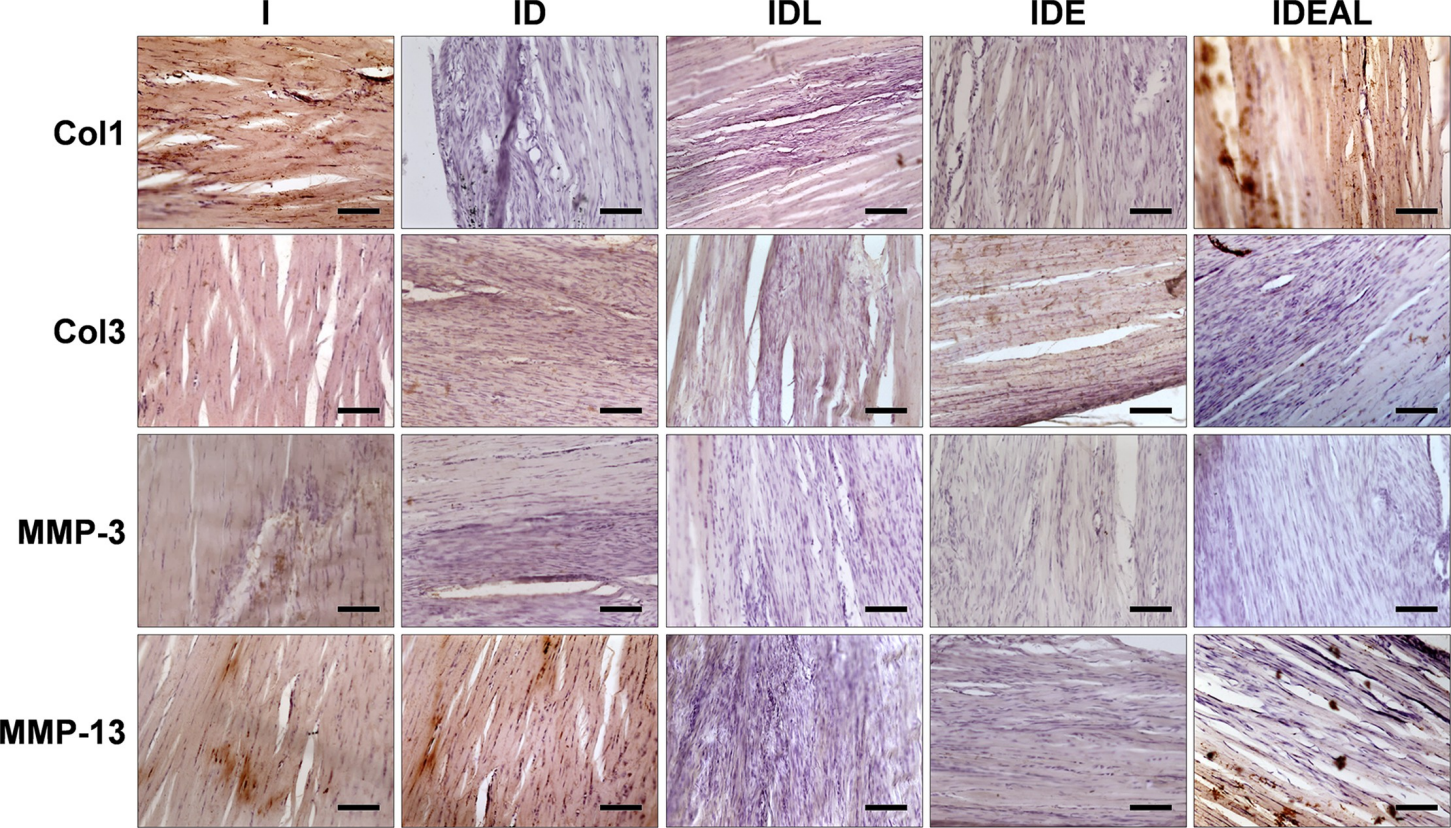
Effects of LPBM and exercise on expression of Col1, Col3, MMP-3 and MMP-13 AT proteins. I, injured (n = 4); ID, injured diabetic (n = 4); IDL, injured diabetic plus LPBM (n = 4); IDE, injured diabetic plus aerobic exercise (n = 4); IDEAL, injured diabetic plus aerobic exercise and LPBM (n = 4). ^a^P<0.05 vs I group; ^b^P<0.05 vs ID group; ^c^P<0.05 vs IDL group; ^d^P<0.05 vs IDE group. Original magnification is x200 and bar is 100 μm.

Table 2 displays the variability of the immunohistochemical scores for Col1, Col3, MMP-3 and MMP-13 regarding each image sample and experimental groups. Different levels of Col1 immunoreactivity were observed in the ECM of all groups with the exception of ID, which presented no expression of Col1 in comparison with I (P < 0.05). Conversely, IDEAL presented stronger Col1 immunoreactivity when compared to ID (P < 0.05). Considering Col3 expression, IDEAL exhibited weaker Col3 immunoreactivity than IDE (P < 0.05). Both IDL and IDEAL demonstrated weaker expression of MMP-3 in comparison to I (P < 0.05), while IDL presented no expression of MMP-13 when compared to ID (P < 0.05). Fig 2 shows a panel with representative images used in immunohistochemical quantification.

**Table 2:** Variability of immunohistochemical scores (n) for Col1, Col3, MMP-3 and MMP-13 of the AT micrographs between groups.

Protein expression	Groups				
	I	ID	IDL	IDE	IDEAL
Col1					
No expression	0	4	1	0	0
Weak expression	0	0	3	3	0
Moderate expression	2	0	0	1	4
Strong expression	2	0	0	0	0
Median IHC score (IQR)	2.5 (2–3)	0 (0–0) ^a^	1 (0.25–1)	1 (1–1.75)	2 (2–2) ^b^
Col3					
No expression	0	0	0	0	1
Weak expression	1	1	2	0	3
Moderate expression	3	3	2	4	0
Strong expression	0	0	0	0	0
Median IHC score (IQR)	2 (1.25–2)	2 (1.25–2)	1.5 (1–2)	2 (2–2)	1 (0.25–1) ^d^
MMP-3					
No expression	0	0	3	1	3
Weak expression	0	4	1	3	1
Moderate expression	4	0	0	0	0
Strong expression	0	0	0	0	0
Median IHC score (IQR)	2 (2–2)	1 (1–1)	0 (0–0.75) ^a^	1 (0.25–1)	0 (0–0.75) ^a^
MMP-13					
No expression	0	0	4	2	3
Weak expression	2	0	0	2	1
Moderate expression	2	4	0	0	0
Strong expression	0	0	0	0	0
Median IHC score (IQR)	1.5 (1–2)	2 (2–2)	0 (0–0) ^b^	0.5 (0–1)	0 (0–0.75)

I, injured (n = 4); ID, injured diabetic (n = 4); IDL, injured diabetic plus LPBM (n = 4); IDE, injured diabetic plus aerobic exercise (n = 4); IDEAL, injured diabetic plus aerobic exercise and LPBM (n = 4); IQR, interquartile range. (n = 4 per group).

^a^*P* < 0.05 vs I group

^b^*P* < 0.05 vs ID group

^d^*P* < 0.05 vs IDE group.

### Fibroblast number and orientation

**Fig 3:**
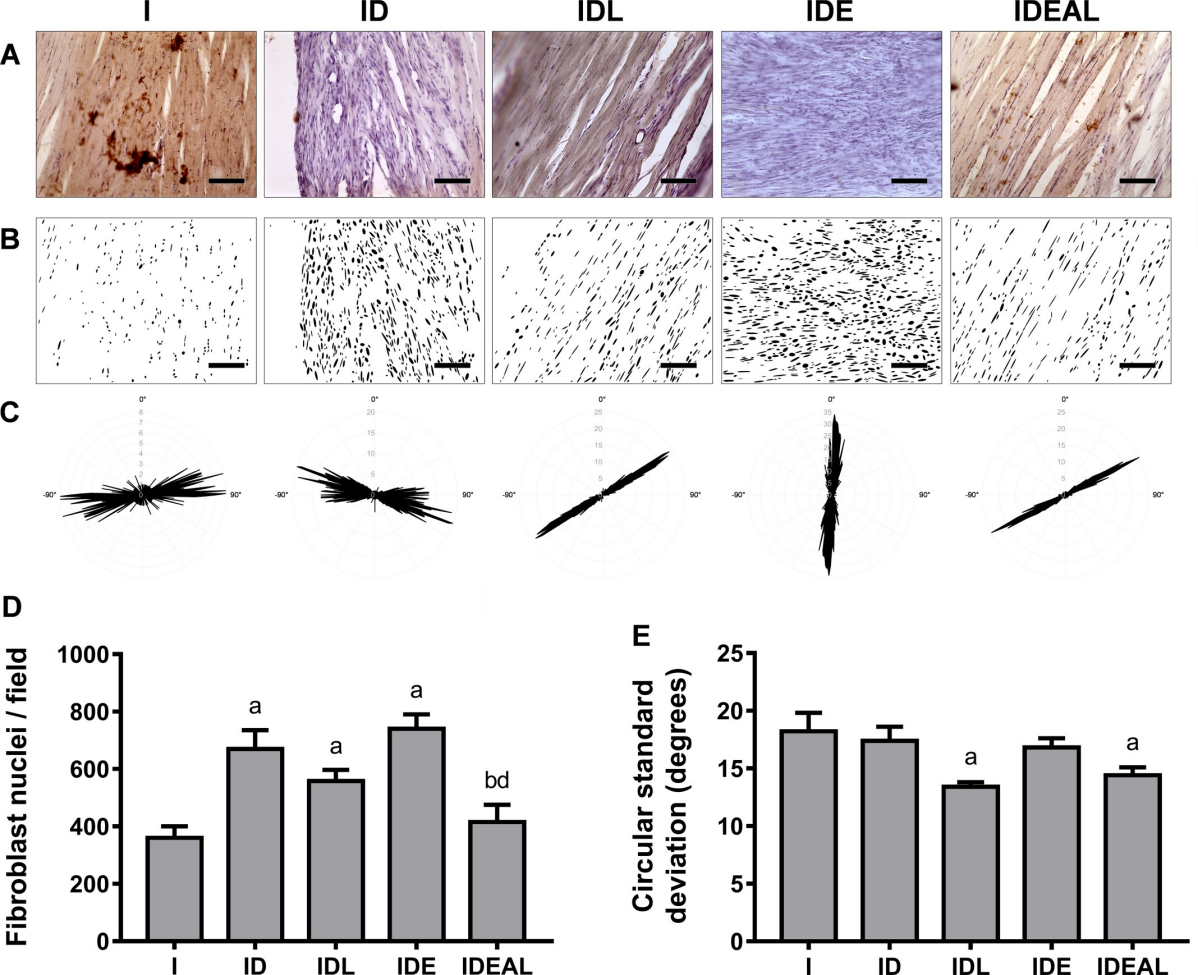
Effects of LPBM and exercise on fibroblast number and orientation in AT. (A) Representative H/DAB-stained images. (B) Binary images containing the segmented nuclei after automated image processing. (C) Representative polar histograms depicting the angular distribution of individual nuclei. The length of the radius of the plot is proportional to angular frequencies. Angles were adjusted for a period of −90° to 90° along the horizontal x-axis. Results of fibroblast nuclei (D) number per histological field and (E) alignment. I, injured (n = 4); ID, injured diabetic (n = 4); IDL, injured diabetic plus LPBM (n = 4); IDE, injured diabetic plus aerobic exercise (n = 4); IDEAL, injured diabetic plus aerobic exercise and LPBM (n = 4). ^a^P<0.05 vs. I group; ^b^P<0.05 vs. ID group; ^c^P<0.05 vs. IDL group; ^d^P<0.05 vs. IDE group. Original magnification is x200 and bar is 100 μm.

By using the particle analysis of the immunohistochemistry images, it was possible to determine the number of fibroblast nuclei and their organization (Fig 3). ID, IDL and IDE showed an increased number of fibroblasts in comparison to I (P < 0.05). On the other hand, IDEAL decreased the number of these cells in comparison to ID and IDE (P < 0.05).

The circular standard deviation data did not show statistical differences between I, ID and IDE (P > 0.05). However, IDL and IDEAL groups had decreased angular dispersion among the fibroblasts when compared to I (Fig 3; P < 0.05).

### mRNA expression data

**Fig 4:**
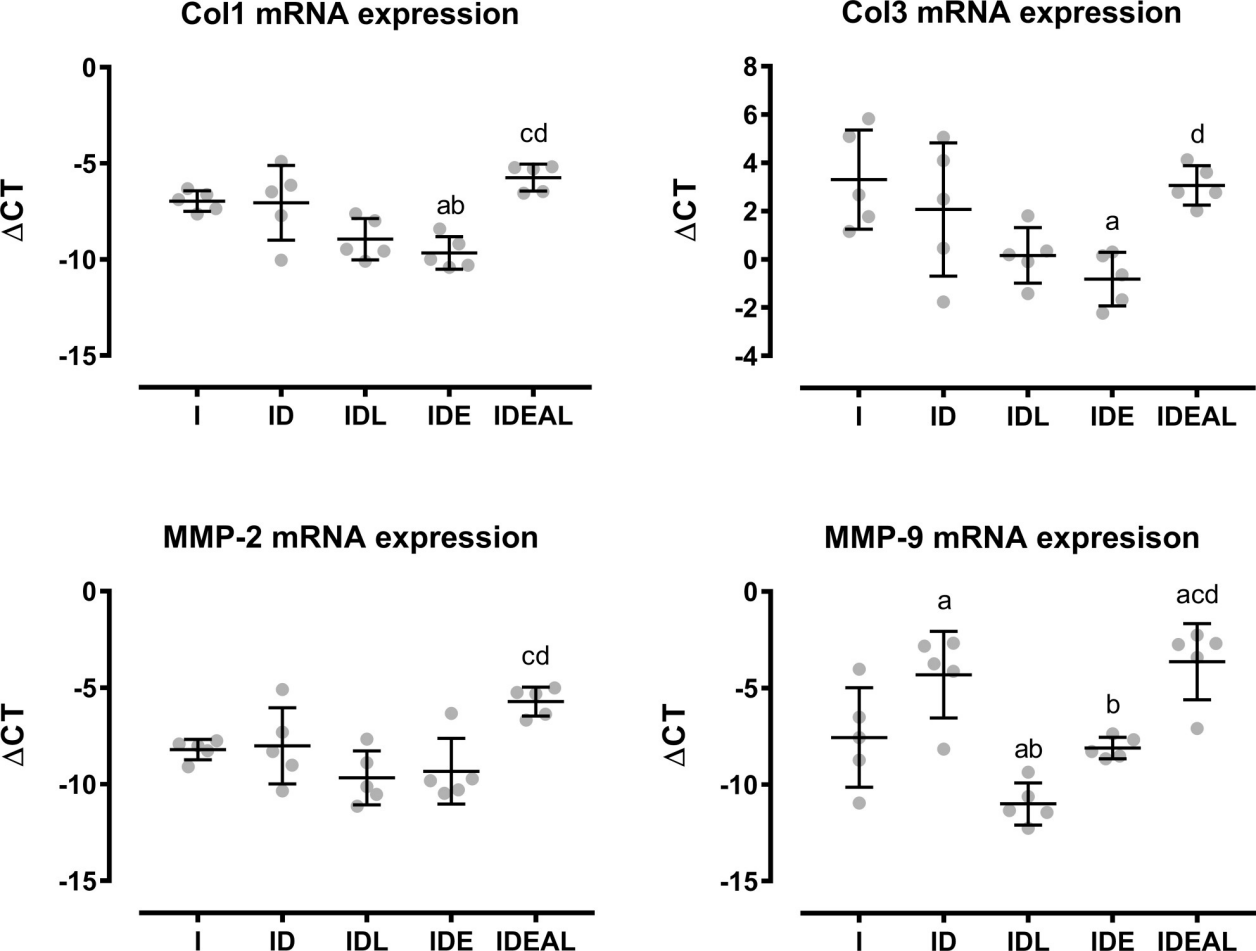
Effects of LPBM and exercise on Col1, Col3, MMP-2 and MMP-9 mRNA expression. I, injured (n = 5); ID, injured diabetic (n = 5); IDL, injured diabetic plus LPBM (n = 5); IDE, injured diabetic plus aerobic exercise (n = 5); IDEAL, injured diabetic plus aerobic exercise and LPBM (n = 5). ^a^P<0.05 vs. I group; ^b^P<0.05 vs. ID group; ^c^P<0.05 vs. IDL group; ^d^P<0.05 vs. IDE group.

The gene expression results are summarized in Fig 4. IDE demonstrated a downregulation in *Col1* mRNA expression in comparison to I and ID (P < 0.05). However, when exercise was combined with LPBM, i.e. IDEAL, *Col1* mRNA expression was upregulated when compared to IDL or IDE alone (P < 0.05). Similarly, IDEAL increased *MMP-2* expression when compared to IDL and IDE (P < 0.05). Considering *Col3* expression, IDE promoted a downregulation in comparison to I, but *Col3* was upregulated in IDEAL when compared to IDE (P < 0.05). *MMP-9* expression was upregulated in IDEAL when compared to I, IDL and IDE (P < 0.05). When compared to I and ID, IDL downregulated *MMP-9* expression, and IDE downregulated *MMP-9* expression when compared to ID (P < 0.05).

## Discussion

Recent literature advocates that DM exhibits an essential role in tendon metabolism and healing. In fact, diabetes has been shown to cause non-enzymatic cross-linking [31] and disorganization of collagen fibers [32], promote inflammatory cell invasion and delayed angiogenesis [33], disrupt neurotrophic and angiotrophic factors [5] as well as matrix protein synthesis and degradation [9], and lead to poor biomechanical properties [9,34,35]. Thus, different strategies have been placed to enhance rehabilitation of tendon disorders in patients with DM [36].

In the present investigation, we focused on the proliferative / early remodeling phase of AT repair by using a rat STZ-induced diabetes model in order to explore the effects of combined LPBM and exercise and compared with the results of either treatment modality alone. This exercise protocol was chosen so that diabetic animals that have an acute tendon injury could participate without any detriment to performance. It is suggested that each intervention has predominant action at different stages of healing. The biostimulatory effect of photobiomodulation is likely to predominate at the initial phases of repair in order to highlight its anti-inflammatory and analgesic properties [37]. On the other hand, when the newly synthesized collagen fibrils start to organize into fiber bundles during late proliferative / early remodeling [38], exercise can provide mechanical transduction and ensure better tendon mechanical properties and collagen orientation [39].

The results of the present study clearly indicate that there is a beneficial interaction between LPBM and exercise and this association improved AT healing if compared to the other diabetic groups or even the non-diabetic group. In a previous work with non-diabetic rats and using the same experimental period, Ng and Fung [24] also found that both LPBM and running were positive to improve load-relaxation, stiffness, and ultimate strength in the AT repair. Here, IDEAL exhibited enhanced ultimate load, absorbed energy, absorbed energy/CSA, ultimate strain, ultimate elongation and ultimate strength. These findings suggest that the interaction between mechanical loading and LPBM augmented tensile strength and energy absorption capacity of the tendons, making the tissue less fragile to rupture. One possible explanation for these mechanical improvements may be due to tendon structural alterations such as enhanced collagen synthesis and re-organization.

The immunohistochemistry data could give suggestions about key matrix proteins involved in early tendon healing. The results of the present study showed that the ID did not exhibit expression of Col1 when compared to the I. The observation that LPBM and exercise together increased the occurrence of Col1 immunoreactivity in the diabetic tendons suggests that this combined treatment modality may be beneficial for early tendon healing. Moreover, the LPBM group played a role in reducing the expression of MMP-3 and MMP-13. MMP-3 works in ECM degradation after mechanical loading without cleaving fibrillar Col1 [40], facilitating directly or indirectly synthesis of ECM components, cell proliferation and differentiation [41]. On the other hand, MMP-13 is able to degrade fibrillar collagens that provide mechanical strength to tissues. In a previous work, Ahmed et al [9] reported that diabetes impairs tendon repair due to a disturbed tissue remodeling involving dysregulated MMP-3 and MMP-13 activity. Here, the immunohistochemistry results indicate an increased turnover of collagen and a net increase of collagen synthesis in diabetic tendons submitted to mechanical loading and laser irradiation, which may be related to the mechanical improvements by IDEAL.

Tissue disorganization has been observed previously in tendons both from diabetic rats [42] and tendons isolated from humans with DM [43]. Here, we used the morphology of the fibroblast nuclei as a feature for evaluating the organization of the healing AT. Indeed, circular standard deviation data indicated that LPBM improved the alignment of the tendon cells in comparison to the other groups. Together with the decreased tendon thickness and cross sectional area, this finding suggests that internal structure of injured tendons experiences optimal repair when exposed to LPBM plus mechanical stimuli. In this way, it seems that photobiomodulation has a central role for collagen orientation since the group exposed to LPBM plus exercise also demonstrated better cellular alignment than IDE. Interestingly, LPBM has been shown to enhance arrangement of the collagen fibers during repair of different tissues such as skin [44]. It is suggested that LPBM acts on cellular events that happen during the inflammatory stage, mainly through reducing pro-inflammatory markers and leading to enhanced fibroblastic activity and collagen production [45–47]. Thus, laser irradiation may result in a faster entry into the proliferative and remodeling phases, which would render a better arrangement of the collagen fibers at the evaluated healing phase of 3 weeks post-injury.

Fibroblasts are elongated cells mainly located between collagen fibers and in the surrounding inter-fascicular space, being responsible for the synthesis and maintenance of the ECM. In the matrix, type I and III collagen represent the most abundant components, making up approximately 90% and 10% of the total collagen content, respectively [48]. While synthesis of type III collagen is essential during the initial stages of tendon repair, it declines as the type I collagen production outbalances and yields to a higher organization of the ECM. The finding that LPBM and exercise together decreased Col3 expression suggests that the combined treatment has shifted the healing phase into a more progressed remodeling phase.

Our results indicate that DM increases the number of fibroblasts. This finding corroborates with previous works that showed that hyperglycemia is able to induce significant changes in cell proliferation and collagen content [32,49]. Also, exercise increased the number of fibroblasts in comparison with LPBM, supporting evidence that moderate mechanical loading enhances anabolic changes via tendon stem/progenitor cell proliferation and tenocyte formation [50]. Interestingly, the combination of LPBM and exercise decreased the number of cells, suggesting normalization of cell proliferation in diabetic tendons.

To hallmark the tendon remodeling at the transcriptional level, we evaluated the expressional changes of *Col1*, *Col3*, *and* both *MMP-2 and MMP-9* which have been associated with tendon remodeling elsewhere [8]. The present results show that the combination of LPBM and mechanical loading upregulated *Col1* and *Col3* mRNA expression after the 3-week experimental model. It should be highlighted that the exercise protocol alone generated downregulation of *Col1 and Col3*, while LPBM alone did not produce significant alterations. So, the combination of both strategies was essential to increase collagen synthesis in diabetic tendon repair and is in line with the reported results on the mechanical parameters. The discrepancy in *Col3* mRNA and protein expression may be due to post-translational modifications of the protein.

The gelatinases MMP-2 and MMP-9 are able to degrade smaller collagen fragments released during activity of the collagenases, although MMP-2 may also influence the collagen remodeling [51]. Previous studies showed that overexpression of *MMP-9* was involved in the molecular mechanisms of tendinopathy in diabetes [8,52]. The results of the present work correspond with the assumption that *MMP-9* is upregulated in diabetic tendons. LPBM alone as well as exercise alone were both able to reduce *MMP-9* mRNA expression. However, the association of both interventions exhibited similar *MMP-9* levels to those of diabetic tendons. Furthermore, upregulation of *MMP-2* in LPBM and exercise groups is also indicative of the ECM remodeling during tendon repair in DM through *MMP-2* and *MMP-9* expression, which would stimulate collagen synthesis in turn. It is however important to emphasize that the increase in these MMPs during the healing process might lead to an augmented ECM degradation and may provide a tendon vulnerable to injury.

It is noteworthy to mention that DM did not produce significant changes on the mechanical parameters in comparison with the non-diabetic group. These findings are in contrast with other studies [25,42], but in line with the work of Volper and co-workers [49] that showed lack of mechanical alterations in acute and chronic STZ-induced diabetes in rats. It is possible that distinct variables such as duration of DM, type of animal strains used and the period of tendon repair analyzed account for the discrepant results. Notably, the type of injury model is a relevant variable that must be highlighted. Different from other studies that investigated the effects of DM on the healing process of completely transected AT [5,34] or by using collagenase [33], we opted for a partial tenotomy to better explore the exercise training during physiological conditions.

A potential limitation of our study was the lack of analyses during several phases of tendon repair. However, since the main aim was to assess the effect of LPBM combined with exercise during proliferative / early remodeling, this design minimized the number of research animals used. Furthermore, the optimal dosage of LPBM in combination with exercise warrants additional studies. Moreover, we could in future researches expand to look at other MMPs and their inhibitors.

In conclusion, this is the first study to explore the effects of exercise combined with LPBM on early AT remodeling in diabetic rats. The results of the current work showed a beneficial interaction of combining both treatment strategies on the biomechanical properties, tissue morphology and the expression of tendon matrix molecules. Further investigations are required to fully understand the underlying molecular mechanisms by which the interaction of mechanical loading and photobiomodulation operates in potentiating healing after soft tissue injuries in DM.

## Supporting information

S1 Fig The AT partial tenotomy injury model with an 18-gauge needle. (TIF)

S2 Fig The moderate aerobic exercise protocol in a motorized treadmill. (MP4)
