# Supplementary figures and images for: Effect of photobiomodulation and exercise on early remodeling of the Achilles tendon in streptozotocin-induced diabetic rats
Source: PLoS One. 2019 Feb 4;14(2):e0211643. doi: 10.1371/journal.pone.0211643 (PMC6361457; doi:10.1371/journal.pone.0211643)

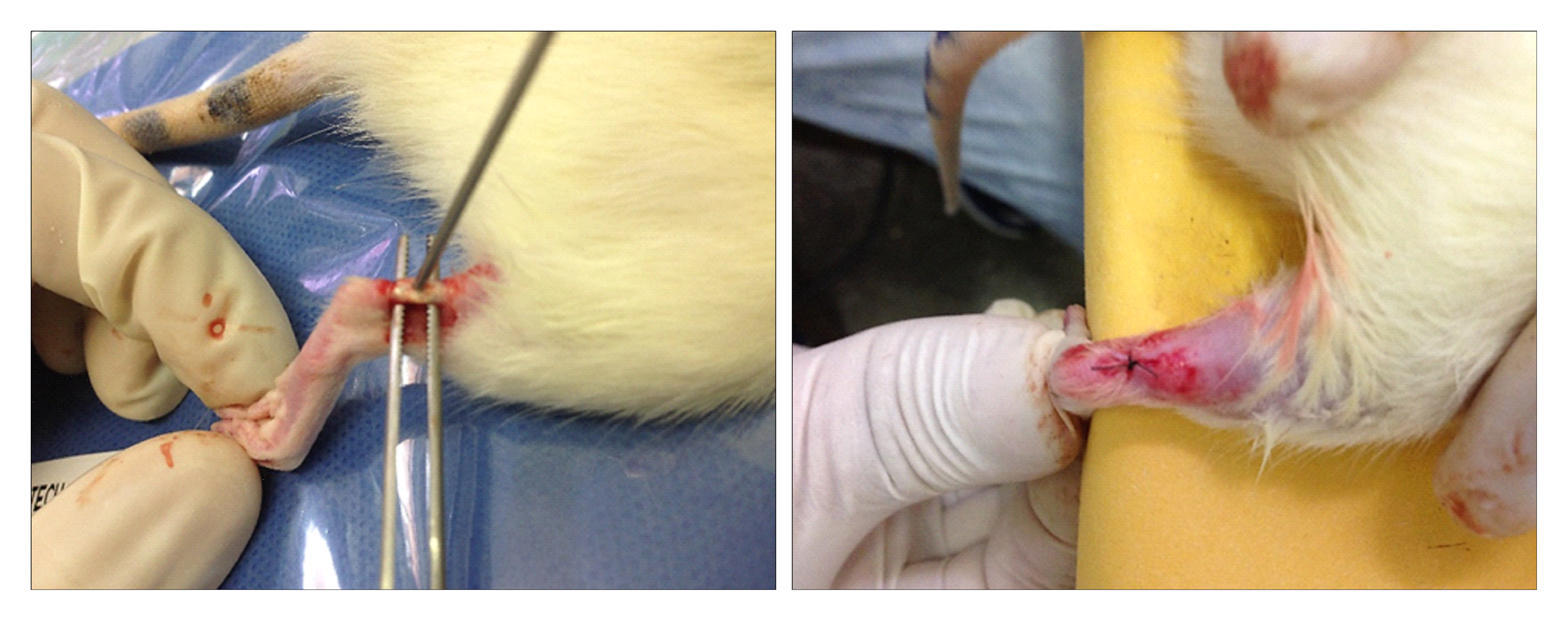

Supplement: S1 Fig — (TIF) [file pone.0211643.s001.tif]
